# Global warming and coastal protected areas: A study on phytoplankton abundance and sea surface temperature in different regions of the Brazilian South Atlantic Coastal Ocean

**DOI:** 10.1002/ece3.11724

**Published:** 2024-08-07

**Authors:** Carolina da Silveira Bueno, Adina Paytan, Cassiano Dias de Souza, Telma Teixeira Franco

**Affiliations:** ^1^ Earth and Planetary Sciences, Ocean Sciences Departament, Institute of Marine Sciences University of California Santa Cruz California United States; ^2^ Interdisciplinary Center of Energy Planning Universidade Estadual de Campinas Campinas Brazil; ^3^ Department of Climate and Environment Federal Institute of Education, Science and Technology of Santa Catarina Florianopolis Brazil; ^4^ Institute of Geosciences Universidade Estadual de Campinas Campinas Brazil; ^5^ Faculty of Chemical Engineering & Interdisciplinary Center of Energy Planning Universidade Estadual de Campinas Campinas Brazil

**Keywords:** coastal protected areas, localized climate change impacts, phytoplankton abundance, sea surface temperature, South Atlantic Ocean

## Abstract

In this study, we examined the relationship between sea surface temperature (SST) and phytoplankton abundance in coastal regions of the Brazilian South Atlantic: São Paulo, Paraná, and Santa Catarina, and the Protection Area of Southern right whales (*Eubalaena australis*) in Santa Catarina (APA), a conservation zone established along 130 km of coastline. Using SST and chlorophyll‐*a* (Chl‐*a*) data from 2002 to 2023, we found significant differences in SST between the regions, with São Paulo having the highest SST, followed by Paraná and Santa Catarina. All locations showed a consistent increase in SST over the years, with North Santa Catarina, APA and São Paulo experiencing the lowest rate of increase. Correlation analyses between SST and Chl‐*a* revealed a stronger inverse relationship in North Santa Catarina and APA, indicating an increased response of Chl‐*a* to SST variations in this region. The presence of protected area appears to play an essential role in reducing the negative impacts of increasing SST. Specifically, while there is a wealth of research on the consequences of global warming on diverse coastal and oceanic areas, heterogeneity among different settings persists and the causes for this necessitating attention. Our findings have implications for both localized scientific approaches and broader climate policies, emphasizing the importance of considering coastal ecosystem resilience to climate change in future conservation and adaptation strategies.

## Graphical abstract

The Baleia Franca Environmental Protection Area spans 3872 km^2^, featuring extensive preserved coastal and marine zones in Brazil's South Atlantic. Preliminary satellite data indicate that this region might have experienced a slightly lower rate of sea surface temperature (SST) change over recent decades compared to neighboring states. These results suggest that the preserved coastal landscape could play a role, warranting further studies to explore whether protected areas can influence SST trends.

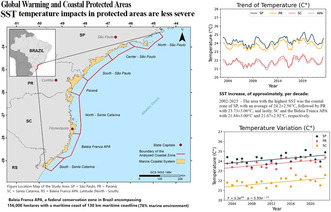

## 1 INTRODUCTION

Global warming represents one of the greatest environmental challenges faced by humanity today, with significant impacts on marine ecosystems. Protected coastal and marine areas play a crucial role in biodiversity conservation and in mitigating the impacts of these climate changes. With the increase in global temperatures, coastal areas are facing significant challenges, highlighting the importance of understanding how these ecosystems are responding to environmental changes.

Remote sensing (RS) has emerged as a highly valuable tool in environmental studies due to its cost‐effectiveness and ability to acquire spatial and temporal data (Mathew et al., 2016). Growing concerns about the effects of climate change have emphasized the need for high resolution temporal analyses to understand climate change the impacts such as increasing sea surface temperature (SST) on various components of ecosystems (Zechiel & Chiao, 2021), such as responses of vegetation composition and productivity (Schucknecht et al., 2013), abundance and evolution of marine biota (Alfonso et al., 2022) and more.

In prior studies (Astor et al., 2013; Goela et al., 2016; Lima & Wethey, 2012; Nicastro et al., 2013), researchers employed decomposition and linear regression techniques to investigate SST changes over time at different sites. For instance, Lima and Wethey (2012), analyzing 19,276 coastal zones, found that approximately 71.6% of these locations exhibited a significant increase in SST at a rate of 0.25 ± 0.13°C per decade, a result similar to that reported by Nicastro et al. (2013) for the Iberian Peninsula (0.2°C per decade). Goela et al. (2016) identified a 0.15 ± 0.05°C increase per decade in the coastal zone of Portugal, while Martínez et al. (2023) reported a 0.36°C increase per decade (annual trend of 0.036°C) in the Gulf of California region. In the North Pacific, SST during summer increased by 0.4°C between 1980 and 2000 (Gregg & Conkright, 2002). Padin et al. (2010) observed an increase in SST at a rate of 0.11 ± 0.03°C per year in the equatorial upwelling system of the Atlantic, a result close to that found by Astor et al. (2013), who recorded a 1.13°C increase over 13 years (0.1 ± 0.04°C per year) in the Cariaco Basin near the Venezuelan coast.

At the Brazilian coastline, a study conducted by Paloschi and Noernberg (2021), using in‐situ SST data and SST derived from MODIS (SST NASA MODIS L3) for the period of 1985–2018, identified a trend of temperature increase of 0.024°C per year in the coastal region adjacent to the state of Paraná (25°12′ S–26°0′ S). Similarly, Risaro et al. (2022), in a study encompassing the southern region of the South American continent, observed a SST increase trend of approximately 0.4°C per decade (0.04°C per year) in the south‐southeast region of Brazil (20°0′ S–30°0′ S).

With consensus among studies regarding global SST increase, albeit with variations in the rate of increase across different regions, concerns arise about how the marine ecosystem will respond to this change. Studies have demonstrated a negative correlation between SST and chlorophyll‐a concentration (Chl‐*a*) levels (Gregg et al., 2005; Ji et al., 2018; Martínez et al., 2023). Utilizing data derived from MODIS, VIIRS, SeaWiFs, and OLCI sensors on satellites such as Terra, Martínez et al. (2023) demonstrated a strong inverse correlation (*R* = −.819) between these two parameters in the Gulf of California region. They reported a decrease in Chl‐*a* levels at a rate of 0.012 mg/m^3^/year. This decline in Chl‐*a* levels, coupled with the increase in SST, had previously been documented by a 6‐year time series conducted by Gregg et al. (2005) using data from various global areas. However, the absolute increase and rate of increase of SST are not uniform across all regions, leading to a critical question: Could the protection of coastal marine areas and adjacent upland watersheds reduced SST increases compared to nearby regions?

This study aimed to explore SST changes and impacts on Chl‐*a* in the coastal zones of the South Atlantic region, specifically in São Paulo, Santa Catarina, and Paraná, Brazil. Additionally, we compare these regions to the coastal zone of the APA Baleia Franca (Environmental Protection Area of *Eubalaena australis*) in Santa Catarina, a federal conservation zone established along 130 km of the coast. Here, we use remote sensing products and report trends in SST in different coastal regions, how the SST change relates to upstream land use and the response of phytoplankton to these SST changes.

We hypothesize that despite differences in SST changes among these regions and their distinct ecosystems, the impacts of increasing SST on the coastal zones will show similarity in responses. However, we postulate that due to upstream land‐use restrictions and the presence of marine protected areas, these regions may experience lower increases in SST and hence exhibit more favorable outcomes in terms of impacts on the ecosystem.

This study is based on a broad range of literature that explores the relationships between land‐use changes and SST across different regions of the world. Specifically, we highlight trends and analyses that demonstrate these relationships to emphasize the importance of considering these dynamics when assessing climate impacts. Understanding these interactions is essential to support our question that preserved areas, which have not undergone significant alterations, tend to exhibit better SST conditions. Lee et al. (2011) highlighted the influence of deforestation in tropical South America demonstrating how changes in land use can impact oceanic conditions. Panda and Rath (2022) emphasized the role of urbanization and land use in modifying coastal and marine ecosystems, underlining the interconnectedness between human activities and rainfall patterns, atmospheric convection, and SST. Wang et al. (2021) addressed how changes in coastal land use significantly affect mangrove habitats, which are crucial for a healthy coastal zone. They emphasize the need for land‐use policies that balance mangrove conservation. This reinforces the notion that environmental interventions can play a significant role in modulating SST in the coastal ocean.

An analysis of studies presents a complex narrative about the relationship between SST increase and phytoplankton response as represented by Chl‐*a*. Boyce et al. (2010) observed a global decline in phytoplankton of approximately ~1% of the global median per year in eight out of 10 oceanic regions, linking this long‐term trend to increases in SST that affect ocean vertical stratification and nutrient availability. Similarly, Trombetta et al. (2019) and Gittings et al. (2018) reported a decreasing phytoplankton bloom strength in shallow coastal waters in the Mediterranean and the Red Sea with increasing SST, and Ji et al. (2018) identified such trends in the East China Sea. Fernandes et al. (2012) identified that in the South Atlantic, Meroplankton abundance was positively correlated with SST and negatively with phytoplankton and Oliveira et al. (2021) observed that variations in phytoplankton communities along the Brazilian coast were influenced by SST. Thomalla et al. (2023) observed significant changes in phytoplankton phenological trends, related to climatic factors including SST, while Karnan and Gautham (2024) noted seasonal variation in phytoplankton in the southern Indian Ocean. The study from Haëck et al. (2023) observed how variations in SST influence phytoplankton blooms in the Gulf Stream region and Hidayat et al. (2023) noted that high levels of Chl‐*a* are inversely related to low SST and salinity conditions, and vice versa.

Zhai et al. (2013) associated variations in SST with changes in the composition and abundance of phytoplankton in the Central North Atlantic, while Richardson and Schoeman (2004) reported impacts on plankton ecosystems in the Northeast Atlantic with sea warming associated with an increase in phytoplankton abundance in colder regions and a decrease in warmer regions. In contrast, Liu et al. (2019) observed a negative correlation between phytoplankton biomass and SST in the Yellow Sea. Behrenfeld et al. (2016) emphasize that variations in Chl‐*a* are not simple indicators of biomass, but also reflect physiological adjustments in cellular pigmentation due to changes in light and nutrients. The latter study suggests that contemporary relationships between changes in Chl‐*a* and ocean warming do not necessarily indicate proportional changes in productivity, as light‐induced decreases in Chl‐*a* may be associated with constant or even increased photosynthesis. On the other hand, Moore and Brown (2020), evaluating the impact of SST and light level estimates separately in the surface mixed layer on model performance, found that the addition of SST to abundance‐based models improved the ability to correctly predict phytoplankton composition, with a significant reduction in mean square error. These studies collectively highlight the intricate and regionally specific nature of interactions between SST and Chl‐*a*, underscoring the importance of considering local and global variations, as well as the need for interdisciplinary approaches to understand the implications of these dynamics to marine ecosystems and the global carbon cycle.

The relation between SST and ecosystems response is fundamental to understanding and projecting future trends. While abundant research exists concerning the consequences of global warming on coastal and oceanic systems, the role of protected areas in modulating the effect of global warming on local SST and coastal ecosystems within the protected zone remains an emerging and urgent concern warranting attention. This urgency holds particular relevance for productive systems in coastal zones and their populations. Given that over a quarter of the Brazilian population (26.6%) resides in proximity to the coast (IBGE, 2011), translating to 50.7 million people, it underscores the need to consolidate crucial socio‐economic information pertaining to these regions to understand how populations may be affected from these expected changes to coastal ecosystems they depend on.

## 2 MATERIALS AND METHODS

### 2.1 Study areas

**FIGURE 1:**
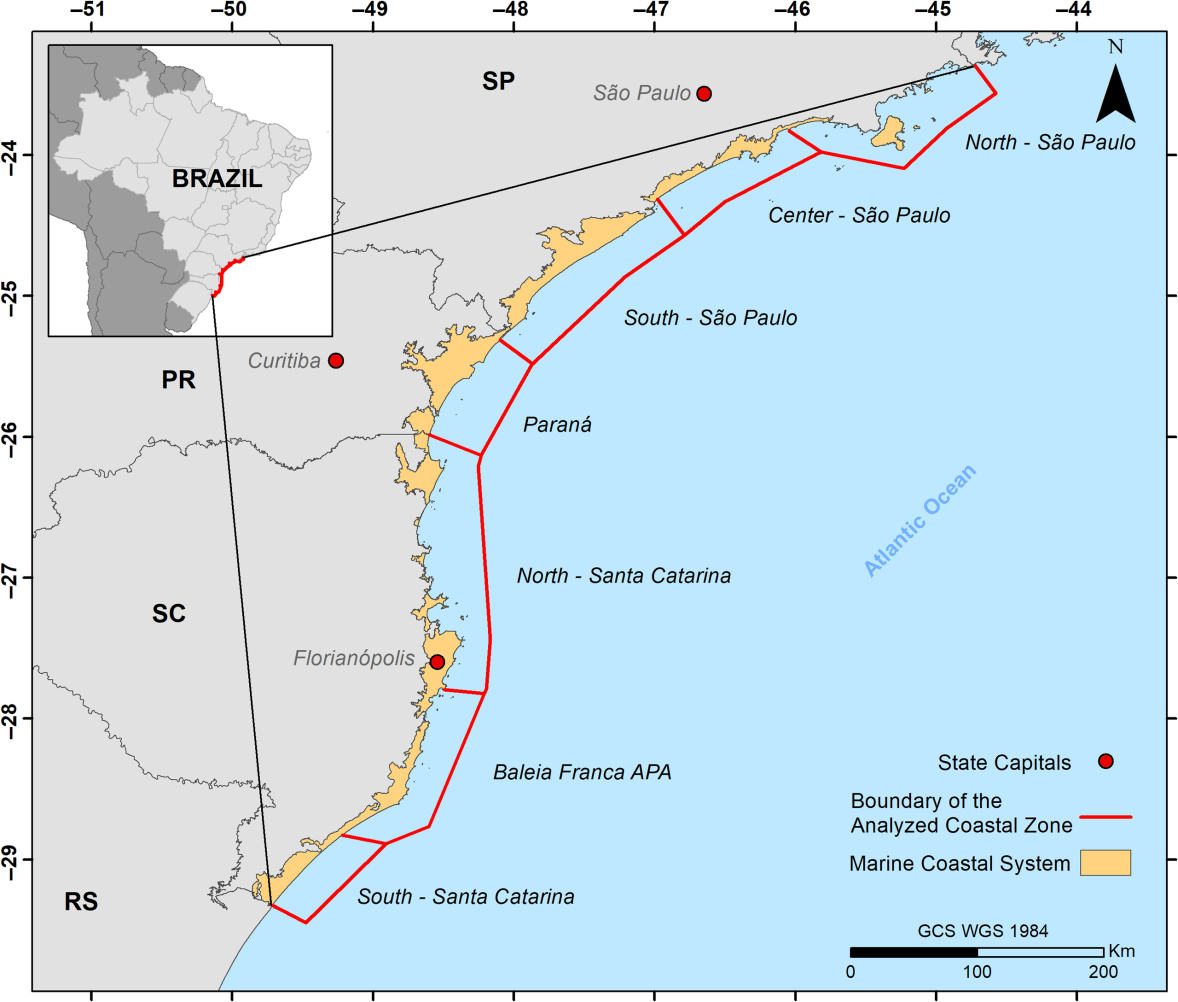
Location map of the study area. SP = São Paulo, PR = Paraná, SC = Santa Catarina, RS = Baleia Franca APA. Latitude (North–South). The latitude ranges within the study area are categorized as follows: North SP (23°35′ S–23°85′ S), Central SP (23°85′ S–24°30′ S), South SP (24°30′ S–25°35′ S), Paraná (25°35′ S–25°95′ S), North SC (25°95′ S–27°80′ S), APA (Right Whale Environmental Protection Area) (27°80′ S–28°80′ S), and South SC (28°80′ S–29°30′ S). Longitude (East–West): Similarly, the longitude divisions are outlined as North SP (44°70′–46°05′), Central SP (46°05′–46°95′), South SP (46°95′–48°10′), Paraná (48°10′–48°60′), North SC (48°60′–48°50′), APA (Baleia Franca Environmental Protection Area) (48°50′–49°20′), and South SC (49°20′–49°75′).

The study area was divided into three sectors corresponding to the coastal areas of the states of Santa Catarina (SC), Paraná (PR), and São Paulo (SP) (Figure 1). Data acquisitions were made within 25 km from the coast (the region between the coast and a 25 km range offshore). The Santos Basin, where the area is located, has a low slope, reaching depths of about 50–60 m at 25 km offshore (Dottori et al., 2023; Figueiredo et al., 2023; Hercos et al., 2023). The study region, with SP in the southeast and PR and SC in the south of Brazil, is primarily located in the Santos Basin. This basin stretches along the coast from São Vicente in SP to north of Florianópolis in SC. South of the Santos Basin is the Pelotas Basin, which extends to the end of SC and continues along the state of Rio Grande do Sul to the border with Uruguay (Lessa et al., 2018).

Due to the extent of the study area and limitations of the Giovanni—NASA platform, the coastal zone of SC and SP states was subdivided into three sub‐sectors for the acquisition of chlorophyll concentration and SST data (Southern Santa Catarina, Environmental Protection Area of *Eubalaena australis*, or Baleia Franca Environmental Protection Area (APA), and Northern Santa Catarina for the state of SC, and Southern São Paulo, Central São Paulo, and Northern São Paulo for the state of SP). Subsequently, the mean of the values of SST and Chl‐*a* was calculated for each region. In total, the analyzed coastal zone covers an area of 29,023 km^2^, with the state of SC representing 12,961 km^2^ (including the APA with 3872 km^2^) of this total, and the states of PR and SP covering 3272 and 12,790 km^2^, respectively.

The surveyed area includes the Environmental Protection Area of Southern right whales (*Eubalaena australis*)—(Baleia Franca APA), a federal protected area in Brazil encompassing 156,000 ha of land with a maritime coastline of 130 km (78% marine environment) and encompasses nine municipalities. The economy within the APA region is diverse, with a notable emphasis on economic activities related to fishing, forestry, and tourism. Fishing is a fundamental part of the economy, with a focus on the production of shrimp and other seafood. The region is historically populated by small communities of artisanal fishermen and support a significant number of families that survive on artisanal fishing (Martins & Dias, 2017).

**FIGURE 2:**
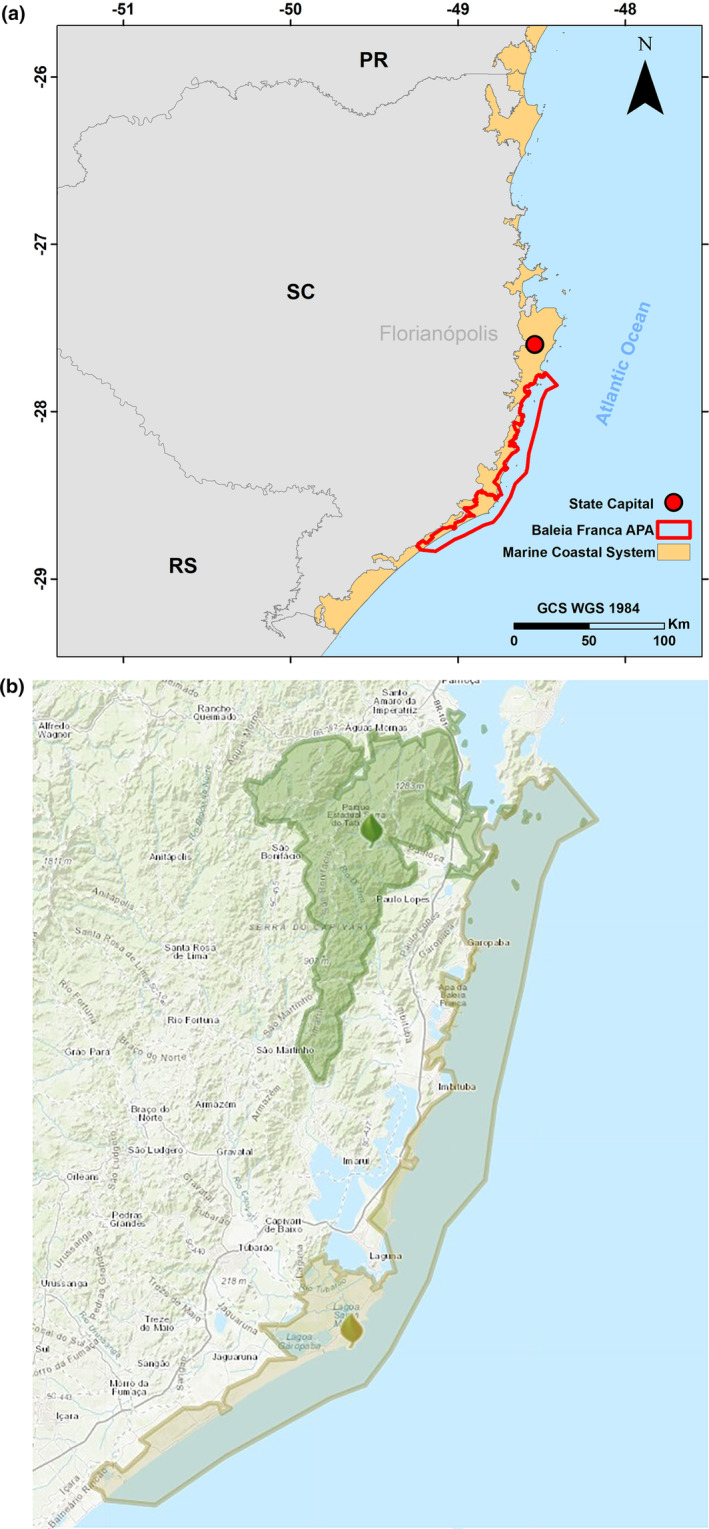
(a) Baleia Franca APA and (b) boverlap with other conservation units of protected area. *Source*: Image (b) is from the Socio‐Environmental Institute, available at: https://uc.socioambiental.org/pt‐br/arp/2581.

The southern right whale population along the Brazilian coast, which has been heavily impacted by whaling until 1973, has since been restricted to southern Brazilian waters. The creation of the APA (Area of Environmental Protection) in 2000 aimed to protect this species and its habitat. The APA serves as a crucial area for the southern right whale's reproduction, with a seasonal presence between May and November. This region is significant for biodiversity and ecological balance, and its proximity to the “Serra do Tabuleiro” conservation unit enhances its conservation impact, as shown in Figure 2 (ICMBIO, 2023; Renault‐Braga et al., 2023).

### 2.2 Data source

The Chl‐*a* and SST products, representing, respectively, the concentration of chlorophyll‐*a* (mg/m^3^) in the ocean's surface and the SST (°C), are derived from MODIS imagens. These data have a spatial resolution of 4 km and a temporal resolution of 8 days, with the temporal series commencing in mid‐2002 to 2023. They are processed by the NASA Ocean Biology Processing Group (OBPG) based on combining the OCx band ratio (OC3/OC4) proposed by O'Reilly and Werdell (2019) with the color index (CI) proposed by Hu et al. (2019), in the case of the Chl‐*a* parameter, and based on algorithms developed by the Rosenstiel School of Marine and Atmospheric Science (RSMAS), in the case of the SST parameter. The data are distributed free of charge via the Ocean Color portal. For this paper, data resulting from processing R2022 (Chl‐*a*) and R2019 (SST) are being used.

### 2.3 Data analysis approach

After the acquisition and quality control of the 8‐days Chl‐*a* and SST data, time series were generated for each of the study areas. Subsequently, descriptive statistics were calculated for each series using Python and/or R programming. SST and Chl‐*a* data covered the period from 2003 to 2022. Trend and seasonality analysis of the data was performed using the time series decomposition technique (Verbesselt et al., 2010) with an additive method, as presented in Equation 1.

(1) $$Y_{t}=T_{t}+S_{t}+R_{t}$$

where *Y*_*t*_ represents the value of the time series at time *t*, *T*_*t*_ denotes the observed trend at time *t*, and *e R*_*t*_ is the noise component at time *t*.

The correlation between Chl‐*a* and SST was calculated using the monthly means of both datasets. Due to the non‐normal distribution of the data, Spearman's correlation (*ρ*) (Akoglu, 2018) was chosen for analysis, with the interpretation of the strength of correlation guided by the following values: *ρ* between 0 and .19 (+ or −) = to no correlation; *ρ* between .20 and .39 (+ or −) = weak correlation; *ρ* between .40 and .69 (+ or −) = moderate correlation; *ρ* between .70 and .89 (+ or −) = strong correlation; *ρ* between .90 and 1.0 (+ or −) = very strong correlation.

Linear regression analysis was conducted based on annual averages of SST values. The differences between the regression lines were tested using the ANCOVA method. Linear regression is a statistical technique widely employed in climate and environmental time series data science, as it relies on the linear relationship between variables to calculate and predict quantitative values (Alfonso et al., 2022; James et al., 2021; Mendenhall et al., 2013; Saleh & Al‐Anzi, 2021; Yao et al., 2023; Zechiel & Chiao, 2021).

## 3 RESULTS

**FIGURE 3:**
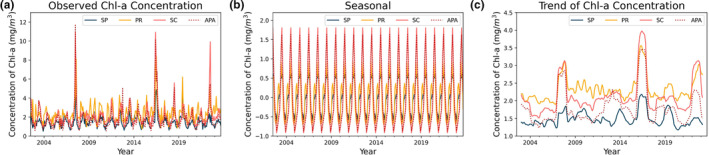
Components generated by the decomposition of the time series of Chl‐*a*.

**FIGURE 4:**
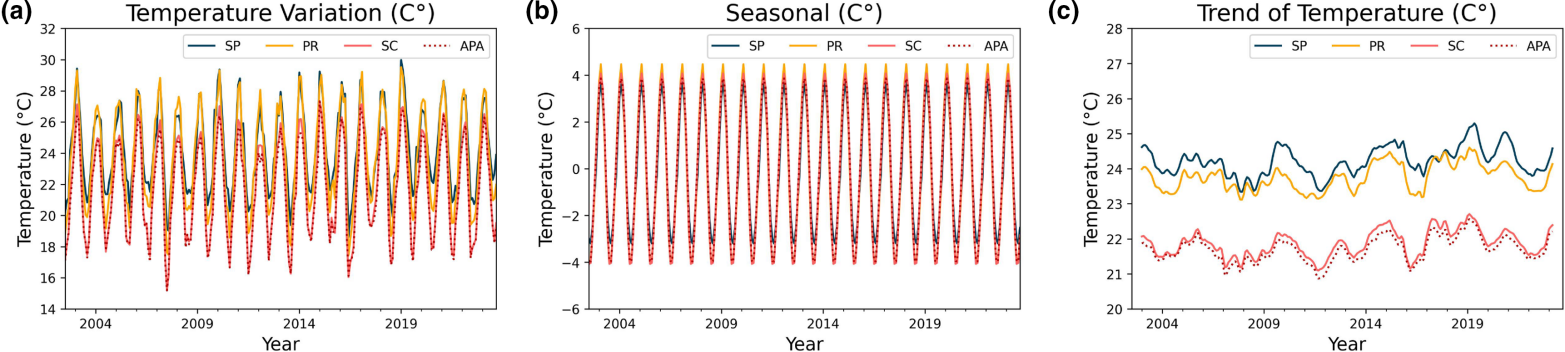
Components generated by the decomposition of the temperature time series.

The seasonal decomposition (Figures 3 and 4) was performed using the “seasonal_decompose” module of the Python library “statsmodels.api” (additive model), which is an excellent tool for visualizing the behavior/seasonality of the data during the analyzed period. Our data revealed well‐defined annual cycles in Chl‐*a* concentration and SST, albeit with differences between coastal areas, as illustrated in Figures 3 (observed chlorophyll concentration, seasonality) and 4 (temperature variation, seasonality), respectively.

Overall, the area with the highest SST was the coastal zone of SP, with an average of 24.2 ± 2.56°C, followed by PR with 23.73 ± 3.06°C, and lastly, SC and the Baleia Franca APA with 21.84 ± 3.00°C and 21.67 ± 2.92°C, respectively. Regarding Chl‐*a* (Figure 3), the area with the highest observed concentration was the coastal zone of Paraná, with an average of 2.33 ± 0.99 mg/m^3^, followed by SP with 1.49 ± 0.64 mg/m^3^, and finally, SC and the Baleia Franca APA with 2.14 ± 1.32 and 1.79 ± 1.27 mg/m^3^, respectively.

It is observed that the coastal zone of Santa Catarina, despite not having the highest overall mean of Chl‐*a* concentration on the surface, presents the highest amplitude of variation in this parameter (Figures 3 and 4, i.e., the highest standard deviation, Table 1). This region also exhibits periodic peaks of anomalous high Chl‐*a* concentration, such as in the years 2008, 2016, and 2022. These peaks do not occur with the same frequency and intensity in other zones, with 2016 being the only year when all three zones simultaneously displayed a marked algae bloom event. Proença et al. (2017) associated this period with a general drop in salinity and triggered by exceptionally intense south‐westerly winds. These winds drove an influx of low‐salinity, nutrient‐rich waters from the La Plata River, located over 1100 km away, combined with high river discharge in the Paraná basin. This extraordinary flux of waters, according to the author, led to the largest outbreak of Diarrhetic Shellfish Poisoning (DSP). Luz and Noenrberg have reached similar conclusion (2022).

**TABLE 1:** Descriptive table for the SST and Chl‐*a* dataset.

Area	Mean	SD	Minimum	25%	50%	75%	Maximum
SST (°C)							
SP	24.20	2.56	18.61	22.11	23.88	26.29	29.97
PR	23.73	3.06	17.43	20.96	23.61	26.49	29.51
SC	21.84	3.00	15.24	19.21	21.79	24.47	27.33
APA	21.67	2.92	15.18	19.18	21.68	24.26	27.32
Chl‐*a* (mg/m^3^)							
SP	1.49	0.64	0.50	1.08	1.43	1.68	6.19
PR	2.33	0.99	0.70	1.75	2.19	2.70	8.40
SC	2.14	1.32	0.78	1.45	1.81	2.32	10.92
APA	1.79	1.27	0.53	1.11	1.51	2.02	11.65

**FIGURE 5:**
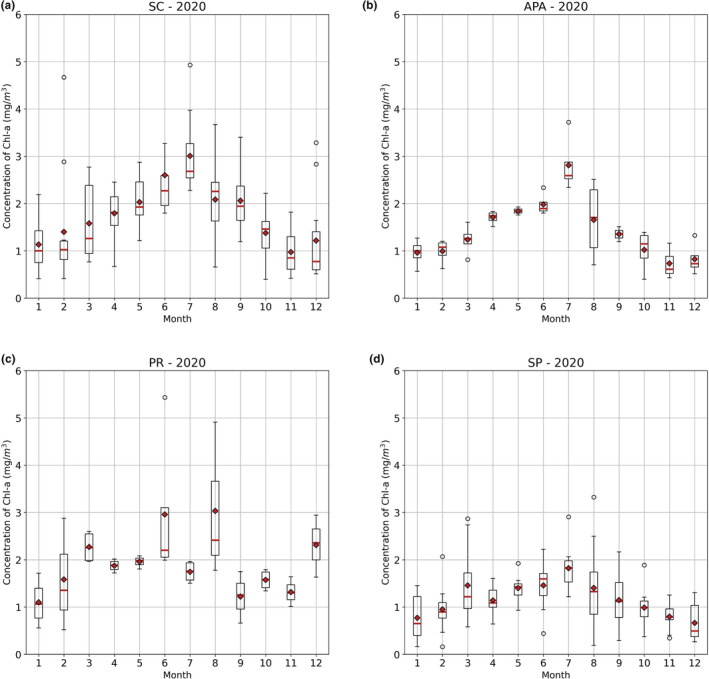
Chlorophyll concentration throughout the year in 2020 for the different studied zones.

**FIGURE 6:**
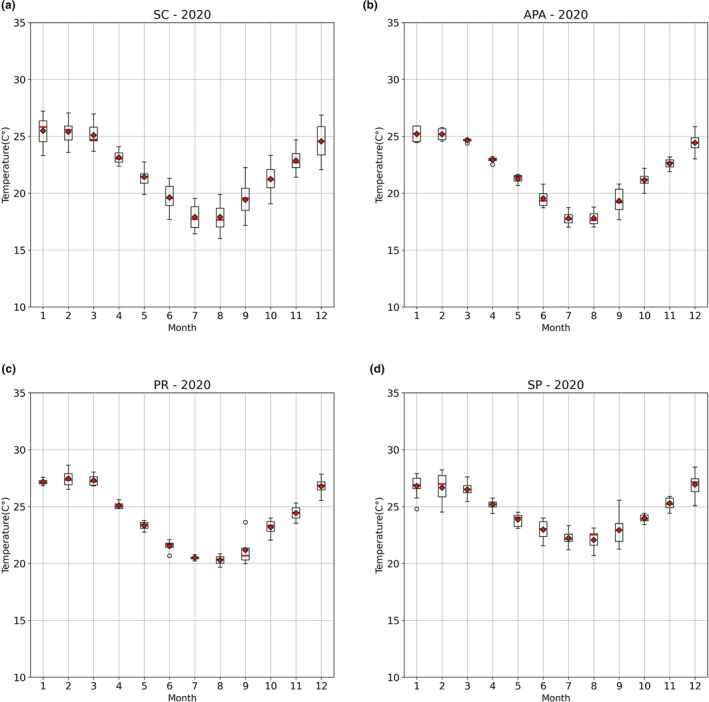
Temperature variation throughout the year in 2020 for the different studied zones.

Figures 5 and 6 display the results of the boxplot. For each year for the two variables studied (Chl‐*a* and SST°C), a boxplot was created by grouping the data for each month of the respective years. The coastal zone of SC also showed a well‐defined annual period of high surface Chl‐*a* concentrations starting in mid‐May, peaking in July–August, and beginning to decline in September (Figure 5). This corresponds to the Southern Hemisphere winter season (Figure 6). Although seasonality is also present in the other zones, the seasonal fluctuations are not as consistent between years.

**FIGURE 7:**
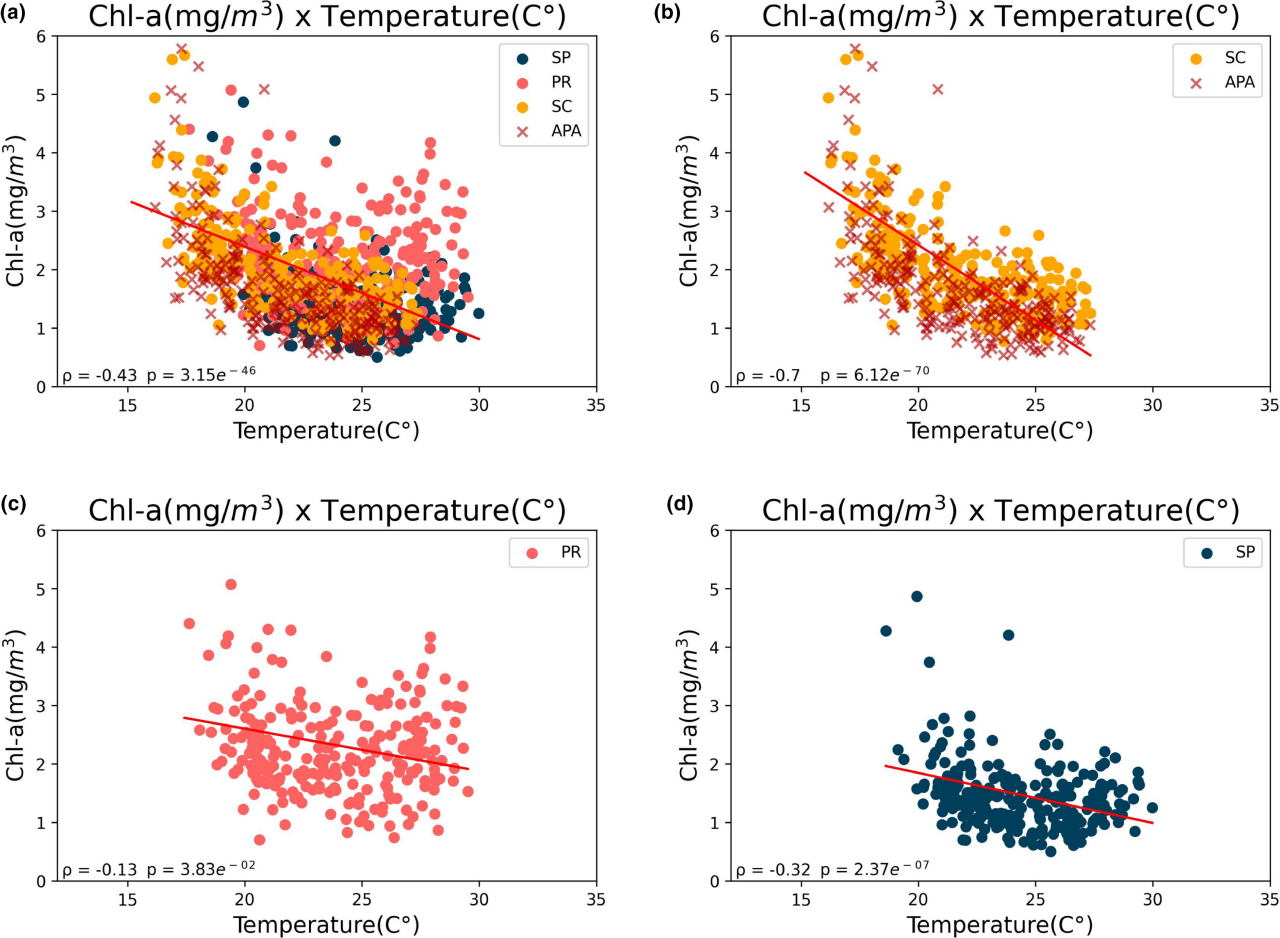
Correlation between SST (°C) and Chl‐*a* (mg/m^3^) data for the entire period considered, 2003–2022. *ρ* = Spearman correlation. (a) Correlation for all areas (SP, PR, SC, and APA). (b) Correlation considering only the areas of SC and APA. (c) Correlation considering only the area of PR. (d) Correlation considering only the area of SP.

The correlation between SST and Chl‐*a* is demonstrated in Figure 7, where a stronger correlation is evident within the coastal zone of SC (*ρ* = −.70) compared to the other regions studied (*ρ* = −.32 and *ρ* = −.13 for SP and PR, respectively).

In Santa Catarina, a strong and negative correlation between these variables suggests that as SST increases, Chl‐*a* concentration substantially decreases. Conversely, SP and PR exhibit weaker correlations, indicating that the relationship between SST and Chl‐*a* in these regions is not as pronounced as in SC. This difference in correlations implies that phytoplankton response to SST, as indicated by Chl‐*a*, can vary considerably among different coastal areas, or there are additional factors unique to each site affecting Chl‐*a*.

For example, studies suggest that the coastal processes regulating phytoplankton communities are influenced by the characteristics of the Brazilian continental shelf. This shelf features significant mixing of waters at the estuary‐ocean interface and exhibits variations in salinity, wind conditions, temperature, and other factors. It showcases diverse climatic, geological, and oceanographic conditions, ranging from temperate to equatorial climates, Precambrian high‐relief metamorphic rocks to Tertiary sedimentary tablelands, and microtides with high wave energy to macrotides with moderate wave energy (Lessa et al., 2018). In the three regions studied, coastal processes affecting phytoplankton communities are influenced by cold water upwelling in summer, the northward spread of Plata Plume Water in winter, and local freshwater discharges (Menezes et al., 2019; Yamashita et al., 2020). In the southeast, the identification of four water masses—Plata Plume Water (PPW), Subtropical Shelf Water (STSW), Shelf Water (SW), and Tropical Water (TW)—highlights their critical role in shaping the composition, distribution, and biomass of phytoplankton groups, as well as nutrient and phosphorus distribution (Lima et al., 2019). In Santa Catarina, the nutrient‐rich South Atlantic Central Waters (SACW), driven by wind, similarly regulate phytoplankton concentrations (Brandini et al., 2014). In São Paulo, the shallow bathymetry and significant tidal mixing result in a bay plume that mixes continental runoff with oceanic water masses, primarily consisting of coastal water (CW), seasonally mixed with Tropical Water (TW), and occasional contributions from South Atlantic Central Water (SACW). These dynamics are further influenced by tides, wind patterns, and freshwater discharges (Carvalho et al., 2014). In Paraná and Santa Catarina, the influence of freshwater discharge and the resuspension of bottom sediments due to physical processes create complex relationships in the concentrations of particulate matter in the water column. The contribution of phytoplankton increases with saline intrusion in Southeastern Brazil Marine Ecoregion (da Cunha Lana et al., 2018; Netto et al., 2018).

**FIGURE 8:**
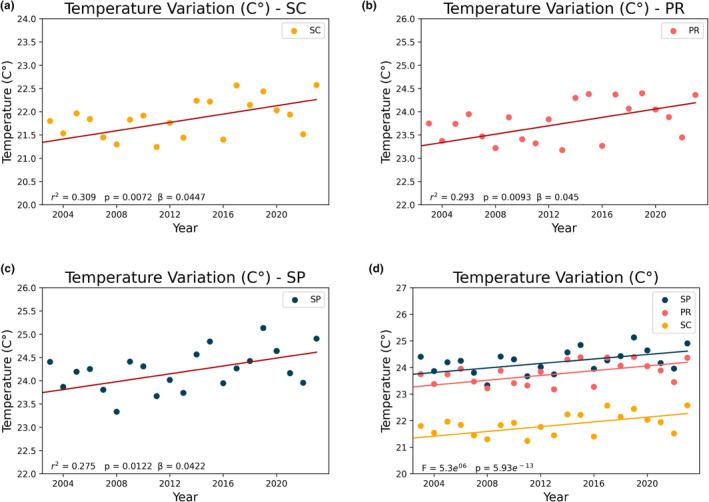
Linear regression of temperature for the states of São Paulo (c), Paraná, (b), Santa Catarina (a). The statistical significance of the difference between the regression lines (d) was calculated using the ANCOVA method, yielding a *p*‐value <.05.

The linear regression analysis demonstrates a consistent increase in SST across all studied coastal areas for the period from 2003 to 2023, albeit with slight variations in the rates of change (Figure 8). From all areas, PR indicate marginally higher annual SST rates of increase, at roughly 0.045°C per year (0.45°C/decade), followed by SC and SP, with an SST rate increase of 0.0447 and 0.0422°C per year (0.447 and 0.422°C per decade), respectively (Figure 8). The APA region exhibits a relatively slower rate of SST increase when compared with the rest of SC state, with an annual rate of increase of 0.0428°C per year (0.428°C per decade).

For the designated analysis period spanning from 2003 to 2023, the dataset reaffirms the overall upward trend in SST for PR and SC, with an SST increase of 0.9 and 0.89°C, respectively, over this time interval. SP and the APA Baleia Franca experienced a slightly lower SST increase of 0.84 and 0.85°C, respectively, over the same period.

### 3.1 Analysis of data exclusively from Santa Catarina (northern portion, APA, and southern region

**FIGURE 9:**
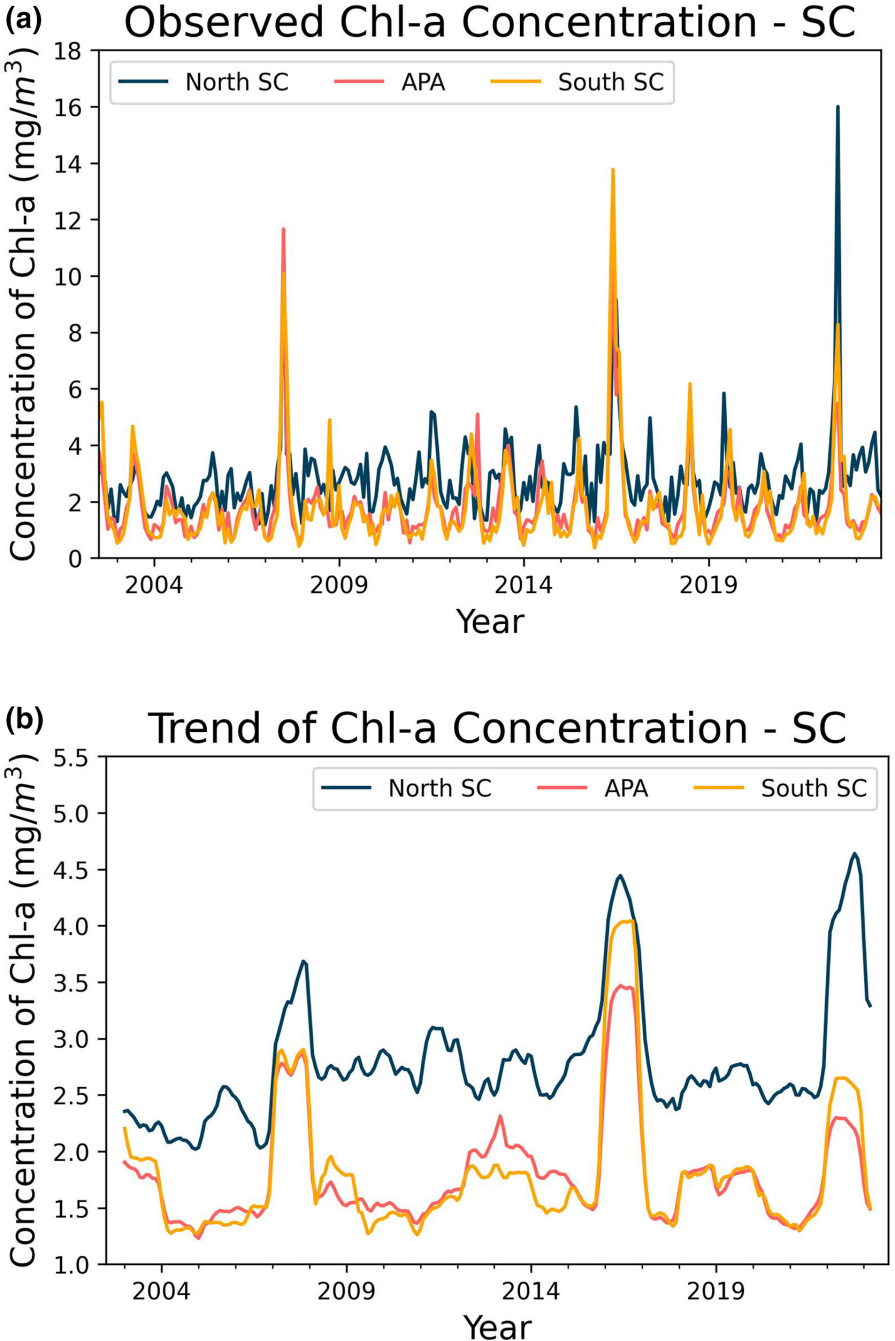
Components generated by the decomposition of the time series of Chl‐*a* (Santa Catarina regions).

**FIGURE 10:**
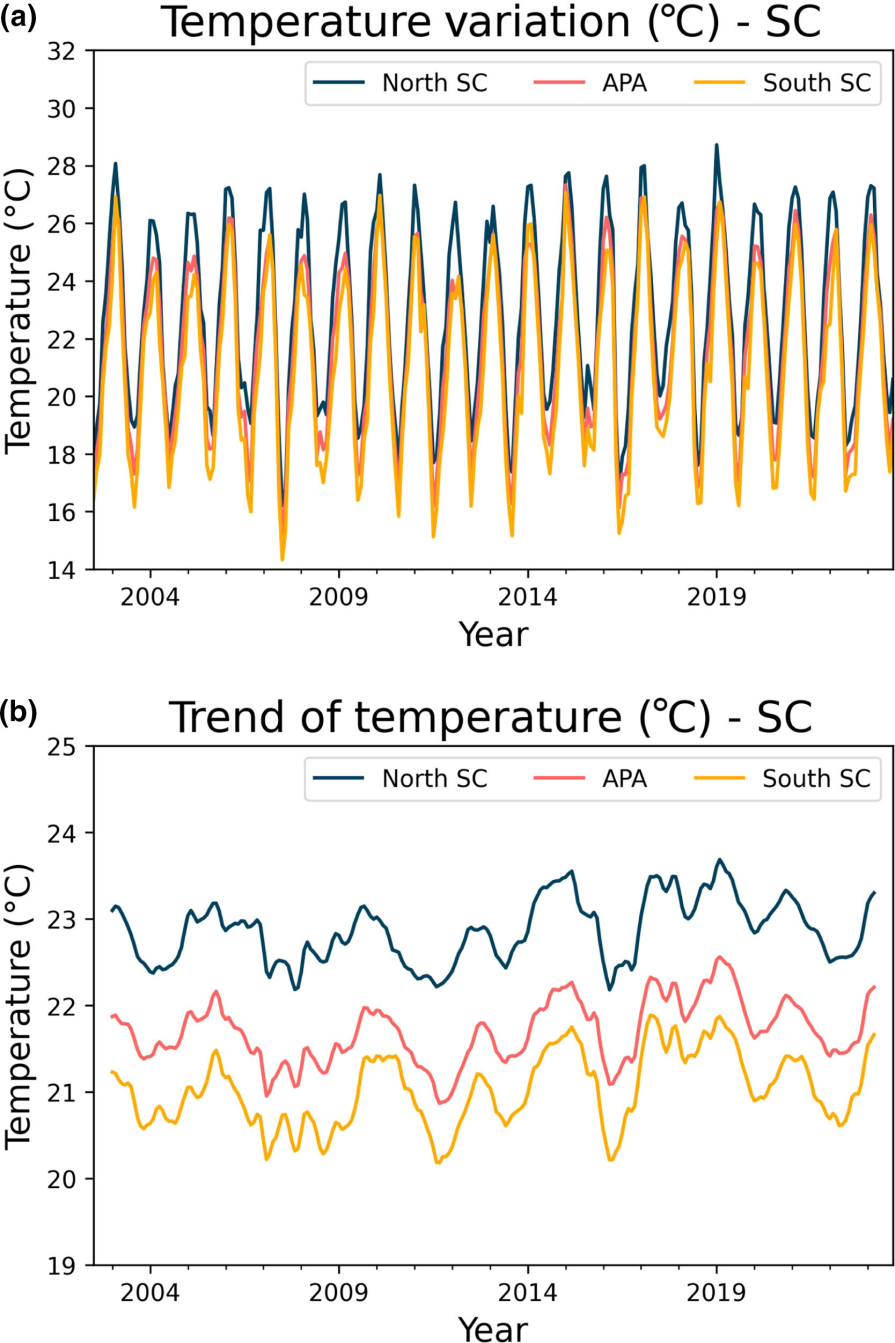
Components generated by the decomposition of the temperature time series (Santa Catarina regions).

**FIGURE 11:**
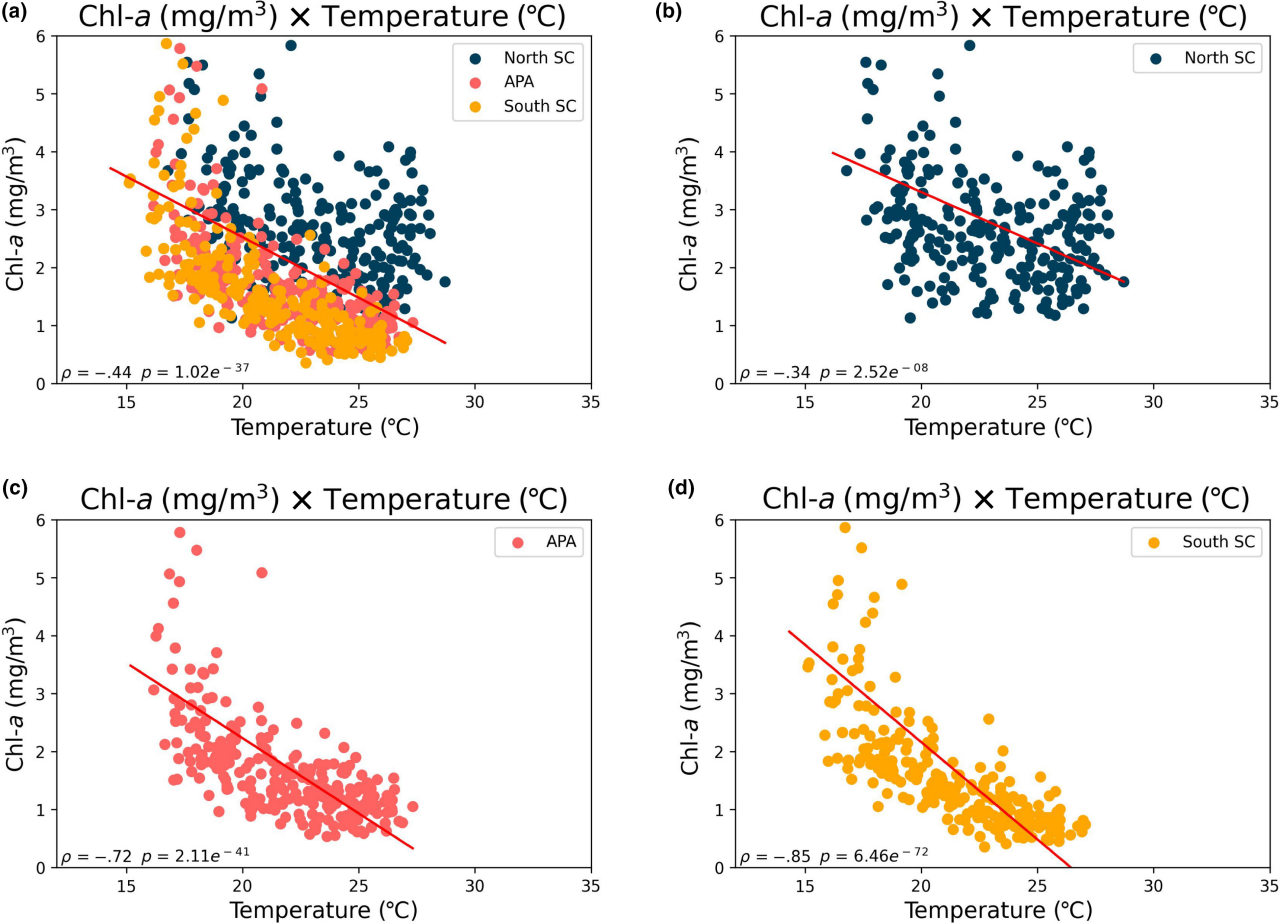
Correlation between SST (°C) and Chl‐*a* (mg/m^3^) data for the entire period considered, 2003–2023. *ρ* = Spearman correlation. (a) Correlation for all SC areas (North SC, APA, and South SC). (b) Correlation taking into account only the area of South SC. (c) Correlation taking into account only the area of APA. (d) Correlation taking into account only the area of South SC.

Overall, all three regions show a similar trend pattern (Figures 9 and 10). However, the northern part has higher average temperatures and chlorophyll concentrations (Table 2). Furthermore, the northern region shows a different correlation level between SST and chlorophyll (Figure 11). The north has a correlation of −.34, while the APA and southern regions have strong correlations of −.72 and −.85, respectively.

**TABLE 2:** Descriptive table for the SST and Chl‐*a* SC‐dataset.

Area	Mean	SD	Minimum	25%	50%	75%	Maximum
SST (°C)							
NORTE	22.80	0.501	21.31	22.47	22.90	23.10	23.53
APA	21.62	0.505	20.07	21.37	21.66	21.88	22.38
SUL	20.95	0.59	19.35	20.60	20.94	21.38	21.90
Chl‐*a* (mg/m^3^)							
NORTE	2.82	0.62	2.11	2.44	2.64	2.89	4.55
APA	1.82	0.51	1.36	1.46	1.62	1.99	3.50
SUL	1.86	0.67	1.27	1.45	1.55	1.88	4.01

**FIGURE 12:**
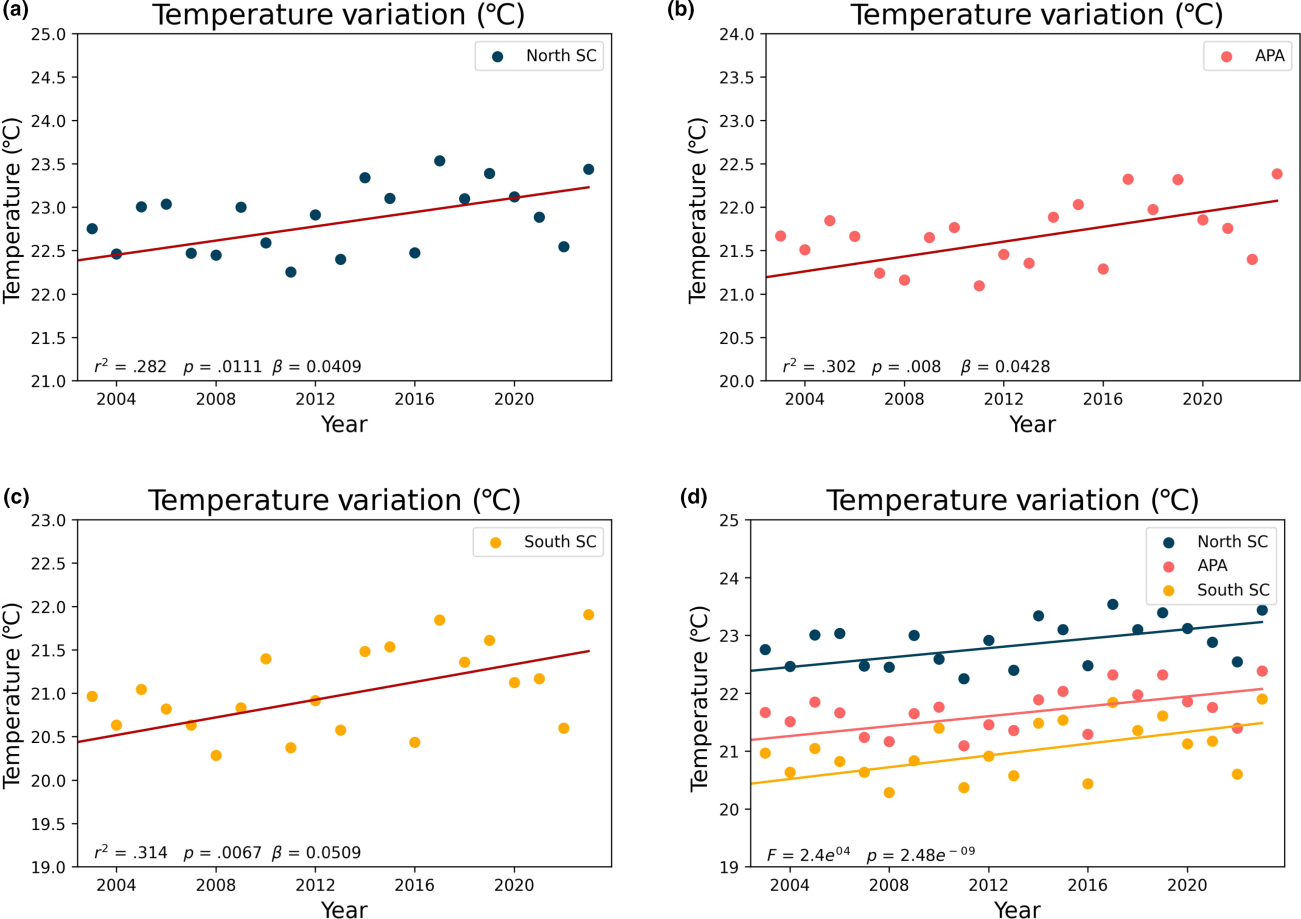
Linear regression of temperature for the SC areas (North SC, APA, and South SC).

When examining linear regression values (Figure 12), the northern region has the lowest increasing trend at 0.0409°C/year, closely followed by the APA region at 0.0428°C/year. The southern region shows a considerably higher trend at 0.0509°C/year. Comparing these regression data with other regions (SP and PR), the northern region of SC has the lowest trend overall, with SP and APA being similar (a difference of 0.0006°C/year), followed by PR and the southern region of SC, which has the highest trend.

## 4 DISCUSSION

The results of our study corroborate several findings from existing literature on changes in SST and Chl‐*a* concentration in coastal regions. In line with previous studies, we found significant differences in SST between the coastal areas of São Paulo, Paraná, and Santa Catarina (Astor et al., 2013; Lima & Wethey, 2012; Nicastro et al., 2013; Serrato & Noernberg, 2019). Specifically, São Paulo exhibited the highest average SST, followed by Paraná and Santa Catarina, which is in line with the findings of Paloschi and Noernberg (2021) and Risaro et al. (2022).

Likewise, we observed a consistent increase in SST over the years in all areas studied, with rates ranging from approximately 0.422°C per decade in São Paulo to 0.5°C per decade in South SC, results that align with previous studies (Goela et al., 2016; Martínez et al., 2023).

Considering the division of the study areas (from north to south: SP, PR, North SC, APA, and South SC), we observe a distinct growth pattern. Analyzing the areas SP, PR, and South SC (which cover the entire north–south extent of the study region), SP showed the lowest growth rate, PR a higher growth rate than SP, and South SC the highest growth rate. This pattern suggests that the growth rate increases as one moves south. However, following this pattern, one would expect North SC and APA, geographically located between PR and South SC, to have growth rates higher than PR but lower than South SC. Contrarily, we observed a substantial decrease in the North SC region and an increase in the APA area, with values similar to those presented in SP. Immediately after APA, there is a significant increase in the South SC region, which showed the highest rate among all studied areas.

These observations indicate that the region between North SC and APA acts as a window of lower growth rate between two areas of higher growth rate. These results underscore the importance of considering specific regional variables when analyzing growth trends and their climatic implications.

Regarding Chl‐*a* concentration, our results showed variations, with Paraná exhibiting the highest concentration, followed by Santa Catarina and São Paulo. These findings are consistent with previous studies that have highlighted the influence of additional factors such as nutrient availability and local oceanographic conditions (Gregg et al., 2005; Ji et al., 2018), as also suggested by Oliveira et al. (2021) for variations in chlorophyll in the South Atlantic region of Brazil. This also suggests that despite the strong relationship between temperature and Chl‐*a* on a seasonal time scale, temperatures may not be the direct driver of the trend, and other factors that co‐vary with temperature and seasonally like irradiance, mixing, and nutrient levels are responsible for the observed correlation.

This can be supported by the literature, which shows that although the use of satellites to study chlorophyll concentration is widely adopted, it remains a complex field of study depending on the study region, with significant methodological challenges due to numerous factors that can influence ocean color. Retrieving information on phytoplankton abundance using bio‐optical methods in water is difficult and requires the development of regional algorithms (Carvalho et al., 2014). Rudorff et al. (2014) emphasize the need to enhance retrieval algorithms to better describe the bio‐optical variability of the oceans in Southern Atlantic and Southeastern Pacific. Yu et al. (2023) highlighted the importance of considering the influence of other optical substances in the remote sensing retrieval of chlorophyll‐a (OCx Chla). Studies such as those by Carvalho et al. (2014) illustrate the complexities of the bio‐optical properties of the inner continental shelf off Santos and their implications for the performance of ocean color algorithms. These studies emphasize the complexity of the dynamics of colored dissolved organic matter (CDOM) and its strong correlation with salinity, influenced by the La Plata River plume, underscoring the challenges in detecting clear trends for the Brazilian continental shelf in regions located in the Santos Basin. In the future, we hope to find better tools or methods, such as the merged approach proposed by Yu et al. (2023) and Carvalho et al. (2014), to identify long‐term trends in our future research.

Furthermore, our results on the seasonality of Chl‐*a* concentration, especially in Santa Catarina, and the inverse correlation between SST and Chl‐*a* agree with previous studies (Gregg & Conkright, 2002; Martínez et al., 2023). Regarding phytoplankton dynamics, our findings corroborate studies that identified interannual to decadal phytoplankton fluctuations or decline in phytoplankton abundance over time with increasing temperature (Boyce et al., 2010; Fernandes et al., 2012).

However, our study has some limitations; specifically, there are various factors that could influence phytoplankton blooms and changes in SST, indicating the influence of other factors like nutrient availability, river runoff, and local oceanographic conditions unique to each coastal area, as identified by Oliveira et al. (2021). Therefore, further research with extended datasets focusing on exploring the mechanisms driving these observed correlations is necessary to fully understand the impacts of climate change on these coastal regions.

Our results are based on a specific ~20‐year period and a limited area, and we did not consider other environmental variables affecting ocean conditions. As noted by Azevedo et al. (2021), the causality of these patterns is still complex and multifaceted. In the future, more data, particularly from other coastal areas with adjacent vegetation, are needed to support our conclusions more comprehensively.

## 5 CONCLUSIONS

Considering the division of study areas (SP, PR, North SC, APA, South SC), a distinct growth pattern is observed. SP has the lowest growth rate, PR is higher than SP, and South SC has the highest. It is important to mention that almost the entire coastal zone of São Paulo has permanent conservation units, covering about 80% of its coastline with the Atlantic Forest biome (Government of the State of São Paulo, 2024). This suggests that the growth rate increases from north to south. However, North SC and APA, located between PR and South SC, do not follow this pattern. North SC shows a significant decrease, APA aligns with SP, and South SC shows a significant increase, the highest among all areas. These observations indicate that the region between North SC and APA serves as a window of lower growth rates between two higher growth rate areas. This highlights the importance of considering specific regional variables when analyzing growth trends and their climatic implications.

In this sense, our study opens up opportunities for broader studies on protected coastal areas, thus creating possibilities for future research to link new environmental variables on land and sea regarding protected areas an adjacent coastal SST. For example, the United States plans to protect 30% of its coastal zones by 2030, demonstrating the importance of this topic. Hence, our study underscores the need for a more detailed examination of this issue, addressing a gap in the literature that links protected areas with SST, presenting a research opportunity.

## AUTHOR CONTRIBUTIONS

**Carolina da Silveira Bueno:** Conceptualization (equal); data curation (equal); formal analysis (equal); funding acquisition (equal); investigation (equal); methodology (equal); project administration (equal); resources (equal); software (equal); supervision (equal); validation (equal); visualization (equal); writing – original draft (equal); writing – review and editing (equal). **Adina Paytan:** Project administration (equal); supervision (equal); validation (equal); visualization (equal); writing – original draft (equal); writing – review and editing (equal). **Cassiano Dias de Souza:** Data curation (equal); formal analysis (equal); methodology (equal); software (equal). **Telma Teixeira Franco:** Project administration (equal); supervision (equal); validation (equal); visualization (equal); writing – review and editing (equal).

## FUNDING INFORMATION

Fundação de Amparo à Pesquisa do Estado de São Paulo (FAPESP n° 2023/08623‐9).

## CONFLICT OF INTEREST STATEMENT

The authors declare that they have no known competing financial interests or personal relationships that could have appeared to influence the work reported in this paper.

## Data Availability

Data supporting Figure 1 were obtained from the Instituto Brasileiro de Geografia e Estatística (https://www.ibge.gov.br/geociencias/cartas‐e‐mapas/bases‐cartograficas‐continuas.html). Data supporting Figure 2 were obtained from the Instituto Socioambiental (https://uc.socioambiental.org/pt‐br/arp/2581). Additional data were derived from public domain resources: (1) Chlorophyll‐*a* data from MODIS (https://www.oceancolour.org/) (2) Sea Surface Temperature (SST) data from NASA MODIS L3 (https://podaac‐tools.jpl.nasa.gov/drive/files/allData/modis/L3/docs/modis_sst.html). The data that support the findings of this study are available from these public domain resources.
